# Combining physical vapor deposition structuration with dealloying for the creation of a highly efficient SERS platform

**DOI:** 10.3762/bjnano.14.10

**Published:** 2023-01-11

**Authors:** Adrien Chauvin, Walter Puglisi, Damien Thiry, Cristina Satriano, Rony Snyders, Carla Bittencourt

**Affiliations:** 1 Plasma-Surface Interaction Chemistry, University of Mons, 23 Place du Parc, 7000 Mons, Belgium; 2 Chemistry of Surfaces, Interfaces and Nanomaterials, Faculty of Sciences, Université libre de Bruxelles, 50 Avenue F.D. Roosevelt, 1050 Brussels, Belgium; 3 Nano Hybrid BioInterfaces Lab (NHBIL), Department of Chemical Sciences, University of Catania, viale Andrea Doria, 6, 95125 Catania, Italy; 4 Materia Nova Research Center, 3 avenue Nicolas Copernic, 7000 Mons, Belgium

**Keywords:** dealloying, magnetron sputtering, nanoporous thin film, nanostructuring, SERS

## Abstract

Nanostructured noble metal thin films are highly studied for their interesting plasmonic properties. The latter can be effectively used for the detection of small and highly diluted molecules by the surface-enhanced Raman scattering (SERS) effect. Regardless of impressive detection limits achieved, synthesis complexity and the high cost of gold restrict its use in devices. Here, we report on a novel two-step approach that combines the deposition of a silver–aluminum thin film with dealloying to design and fabricate efficient SERS platforms. The magnetron sputtering technique was used for the deposition of the alloy thin film to be dealloyed. After dealloying, the resulting silver nanoporous structures revealed two degrees of porosity: macroporosity, associated to the initial alloy morphology, and nanoporosity, related to the dealloying step. The resulting nanoporous columnar structure was finely optimized by tuning deposition (i.e., the alloy chemical composition) and dealloying (i.e., dealloying media) parameters to reach the best SERS properties. These are reported for samples dealloyed in HCl and with 30 atom % of silver at the initial state with a detection limit down to 10^−10^ mol·L^−1^ for a solution of rhodamine B.

## Introduction

Pollutant residues are strictly regulated in most countries to ensure water and food safety. In this context, there is an increasing demand for pollutant analysis tools with practical and cost-efficient methods. Compared to in-lab standard methods used for pollutant analysis (i.e., chromatography and mass spectrometry), surface-enhanced Raman scattering (SERS)-based sensors have emerged as important candidates due to their rapidity, portability, and cost-effectiveness [1–2]. These SERS sensors are promising for various applications in chemical (e.g., explosive [3] or chemical warfare agents [4]) or biological (e.g., lipid or protein [5]) sensing, environmental monitoring [6] as well as in food safety through the detection of pollutants such as phenol [3,7] or rhodamine [8].

The SERS detection properties are mostly observed in noble metal nanoparticles [2,9–10]. Allowed by their localized surface plasmon resonance (LSPR) in the visible region, silver and gold are the most used materials for the preparation of SERS substrates [11–12]. Although Ag has a higher surface plasmon efficiency compared to that of Au, Ag nanoparticles (NPs) are prone to oxidation. Moreover, they are less thermodynamically stable leading to morphology variation and ultimately to deterioration of their SERS efficiency [13–14]. Besides that, most studies report on the high SERS properties for NPs in suspension [15]. However, NPs suspensions are hard to handle, suffer from poor stability, and can hardly be reused [6]. In this perspective, major interest has been devoted to developing solid SERS platforms made of nanostructured thin films [15–16]. Among them, nanoporous materials show superior properties due to their interconnected nanostructures and large surface areas [17–19]. A myriad of techniques is available for the synthesis of porous nanostructures [20–21]. Among them, dealloying has received particular attention due to its simple methodology [17,22]. This method involves the leaching of the less noble component of an alloy creating a skeleton made of the noble element [23]. Dealloying is usually accomplished through a chemical step in which the alloy is dipped into an etching solution to remove the less noble metal [24]. This process leads to highly homogenous, porous structure substrates with better reliability and stability compared to conventional NP-based SERS substrates [25]. The race towards more efficient SERS platforms has led to the development of highly complex synthesis processes which limits their use in practical applications [15,26–29]. Most reports on efficient nanoporous platforms are based on gold due to their high stability towards oxidation. Therefore, heading towards simple and inexpensive approaches to reach the industrial market turns out to be a necessity.

The origin of the SERS effect relies on the interaction between an intense electromagnetic field and the analyte through an i) electromagnetic enhancement (i.e., ‘hot spot’) and/or a ii) chemical enhancement [10]. Despite the impressive detection limit achieved by the nanoporous structures, little attention has been paid to the sample surface architecture despite of the fact that the SERS effect is highly dependent on the distance between nanostructures [10]. In fact, the electromagnetic enhancement observed between two close NPs or in metallic nanotips exponentially decays when the distance to the metal surface increases [10]. In other words, only analytes that are very close (i.e., less than 3 nm) to the surface experience the electromagnetic field [2]. Moreover, the chemical enhancement occurs at an even shorter effective distance range since the molecules have to bond to the metal surface. Therefore, even though high electromagnetic field enhancement can be achieved using SERS, the resulting signal intensity tends to strongly vary due to surface contamination [30].

In this paper, a simple synthesis method to design bimodal porous silver substrate for SERS is reported. Magnetron co-sputtering of a silver and aluminum target was used for the deposition of the precursor alloy thin film. This approach allows for the synthesis of structured alloy thin films constituted of dispersed alloy columns. The dealloying of these films was implemented in three different media (i.e., hydrochloric acid (HCl), sodium hydroxide (NaOH), and phosphoric acid (H_3_PO_4_)) to highlight their impact on the SERS properties of the nanoporous structure. Using scanning electron microscopy (SEM) and X-ray photoelectron spectroscopy (XPS) the morphology and surface composition of each nanoporous structure were respectively evaluated and used to describe the SERS properties of the samples.

## Results and Discussion

### Morphology of Ag–Al alloy thin films

**Figure 1:**
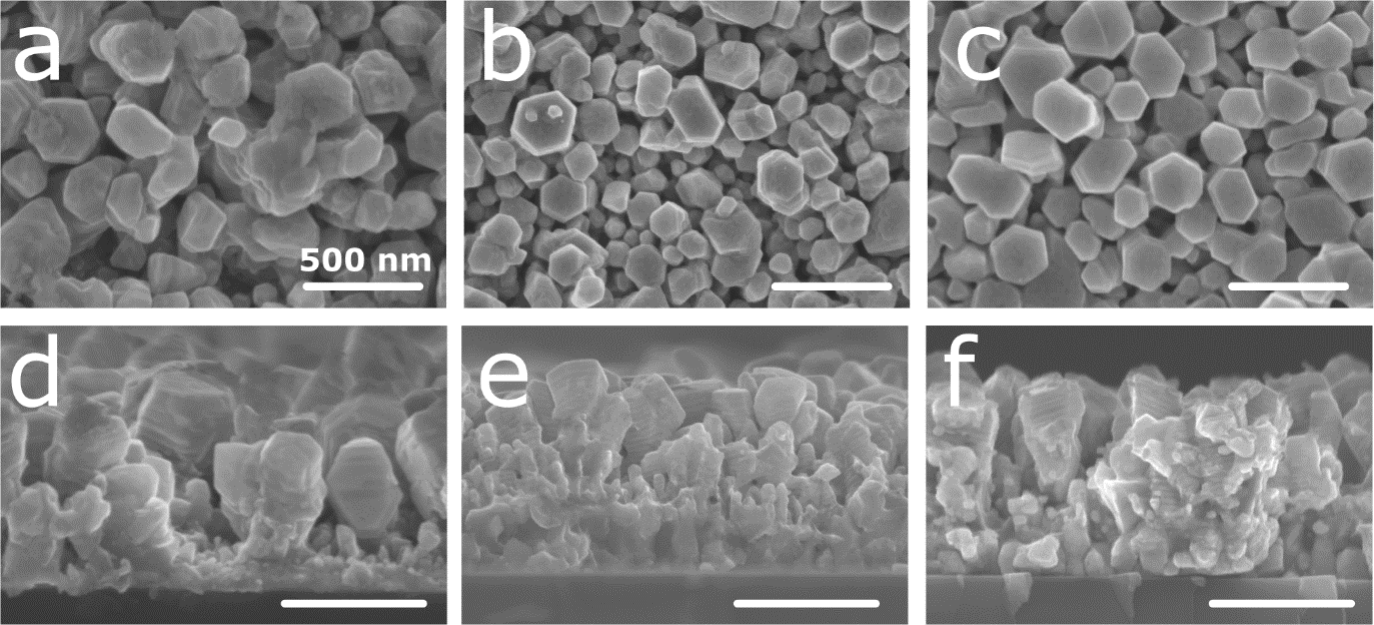
(a–c) Plan view and (d–f) cross-section SEM images of the Ag–Al alloy thin film with an initial composition of (a and d) 18, (b and e) 30, and (c and f) 36 atom % of Ag.

The deposition of silver–aluminum thin films on silicon was accomplished by magnetron co-sputtering using a silver target and an aluminum target. To allow for a good adhesion between silicon and the alloy thin film, a silver adhesion layer was deposited prior to the alloy thin film. Three different silver compositions were selected, namely 18, 30, and 36 atom %, and characterized by SEM/energy-dispersive X-ray spectroscopy (EDX). Figure 1 displays the SEM micrograph of the as-deposited thin films.

The thin films exhibit a columnar morphology (see the cross-section SEM images in Figure 1d–f). The top view images (Figure 1a–c) reveal the presence of dispersed hexagonal columns. A possible explanation for the formation of the hexagonal structure is due to the Guinier–Preston (GP) zone of the silver–aluminum alloy system [31]. The GP zone induces the growth of Ag–Al crystallites in a truncated octahedron shape at low temperatures (below 170 °C) [32–33]. Due to the rather short distance between the substrate and the target (i.e., 10 cm), particles have greater mobility, resulting in the growth of crystalline structures [34]. This induces the faceting of the crystallite and promotes growth in a hexagonal shape. The dispersed column structure can be the consequence of the hexagonal growth and substrate rotation during deposition. Due to the specific geometry of our setup (i.e., the angle between the normal of the substrate and the targets is 30°), the rotation of the substrate induces a shadowing effect. Briefly, the material arriving on the substrate with an oblique angle cannot be homogenously deposited. Thus, a dispersed columnar structure growth is observed [35–36]. For the sample with 18 atom % of Ag (Figure 1a and Figure 1d), the columns are slightly larger and less packed than those in the samples with 30 and 36 atom % of Ag (Figure 1b). Following the aforementioned hypothesis, this observation can be due to the more pronounced effect of the GP zone in the sample with low silver content [31].

### Optimization of the nanoporous structure

#### Influence of the initial Ag content and dealloying time

**Figure 2:**
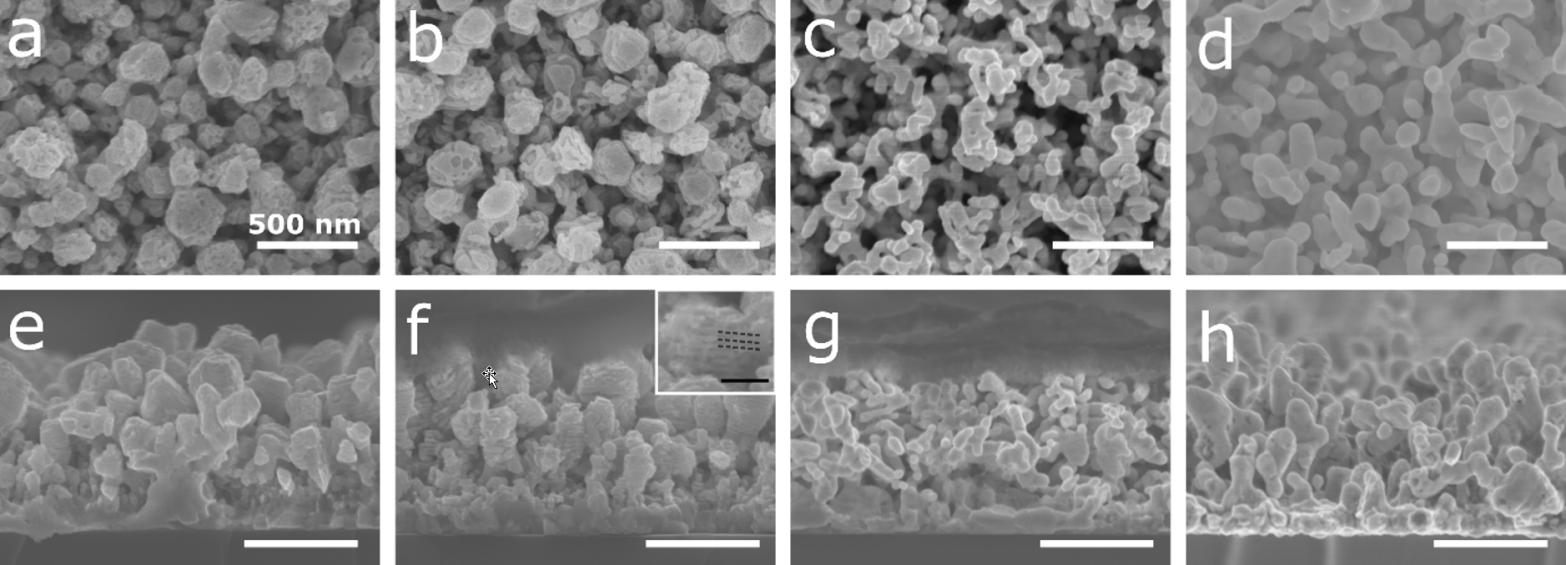
(a–d) Plan view and (e–h) cross-section SEM images of the Ag–Al alloy thin film with an initial composition of 30 atom % of Ag and dealloyed for (a and e) 10, (b and f) 30, (c and g) 60, and (d and h) 120 min in an HCl solution at 1 wt %. Scale bar: 500 nm. Inset of panel (f) corresponds to a magnification of a nanolayered structure, the dashed lines highlight a full layer. Scale bar 100 nm.

The nanoporous structure was tailored to obtain the best SERS efficiency for the detection of rhodamine B (RhB). First, the influence of the dealloying time on the morphology was evaluated. Samples with 30 atom % of Ag were synthesized and the dealloying was performed in HCl for 10, 30, 60, and 120 min. The lamellar structure in the as-prepared sample is due to the rotation of the substrate during deposition. The film presents a multilayer structure composed of Ag-rich and Ag-poor layers which are formed due to distance variation between the rotating substrate and the Ag target (i.e., Ag-rich layers are formed when the substrate is close to the Ag target while Ag-poor layers are formed when the substrate is far from the Ag target). The formation of these nanolayers has already been reported in the literature [24,37]. Higher magnification SEM images of the nanolayers are available in Supporting Information File 1, Figure S1. The SEM micrographs of the dealloyed thin films are shown in Figure 2.

After 10 min in HCl, pores appear on the top of the Ag–Al thin film (Figure 2a) whereas no changes are observed in the cross-section images (Figure 2e). These small pores highlight the early dealloying stage and the propagating front at the grain boundaries [38]. After 30 min in HCl, bigger pores are formed (Figure 2b) and the cross-section image shows structures made of a porous and full layers (Figure 2f). After 60 min of dealloying, the initial structure is hardly observed, and the creation of small ligaments is revealed through the film (Figure 2c and Figure 2g). Finally, after 120 min of dealloying, the material exhibits a nanoporous structure with larger ligaments than those after 60 min of dealloying (Figure 2d and Figure 2h). The increase in the ligament size from 50 to 100 nm and in diameter values between 60 and 120 min, respectively, corresponds to the late stage of dealloying characterized by a step of coalescence of the ligaments [38–39].

**Figure 3:**
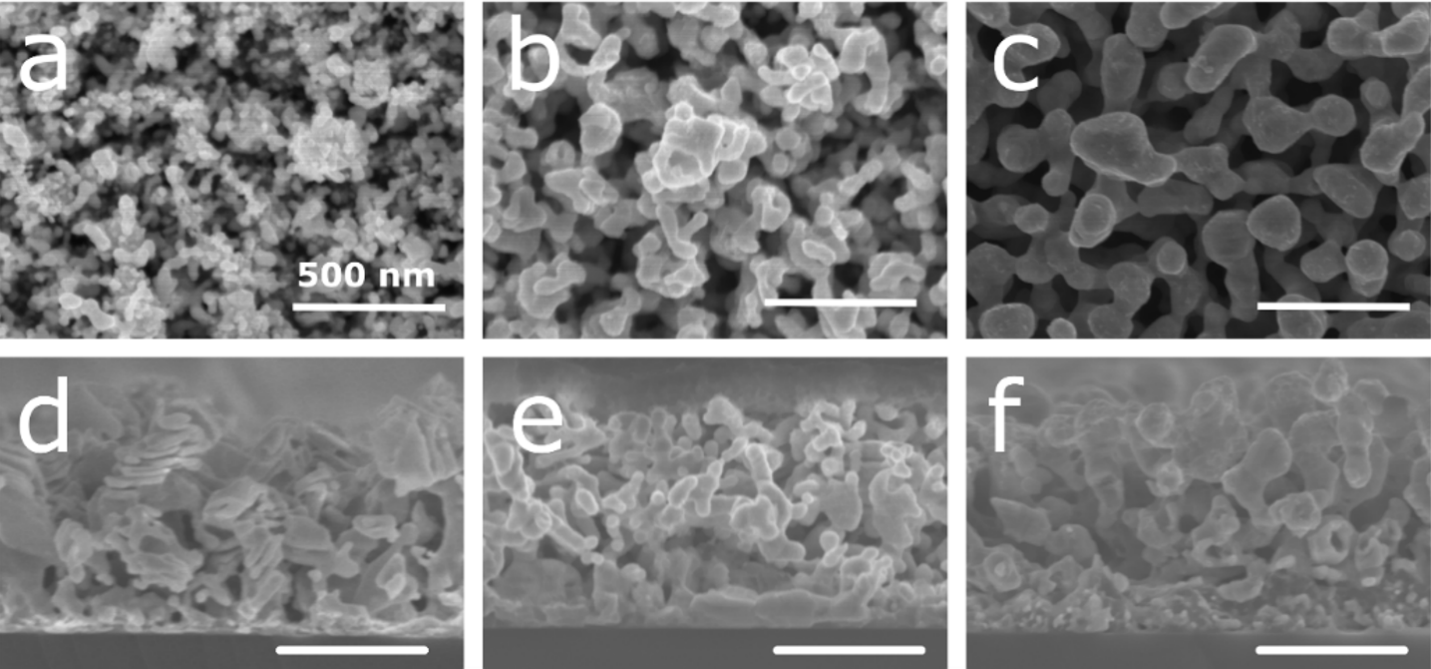
(a–c) Plan view and (d–f) cross-section SEM images of the Ag–Al alloy thin film dealloyed for 60 min in HCl solution at 1 wt % with an initial composition of (a, d) 18, (b, e) 30, and (c, f) 36 atom % of Ag. Scale bar: 500 nm.

Next, the influence of the silver content in the Ag–Al thin film on the dealloyed morphology was studied. For this purpose, the initial alloy composition was tuned at 18, 30, and 36 atom % of Ag. For sake of simplicity, the samples with 18, 30, and 36 atom % of Ag at the initial state will be denoted as AlAg18, AlAg30, and AlAg36, respectively. These concentrations were selected to avoid the passivation of the surface by silver as observed in our previous study (≈50 atom %) [39]. This silver passivation layer is related to the parting limit of the dealloying process, which corresponds to the amount of the less noble material below which dealloying does not proceed [22,40]. The SEM micrographs of samples with different silver content at the initial state and dealloyed for 60 min in HCl are displayed in Figure 3.

**Figure 4:**
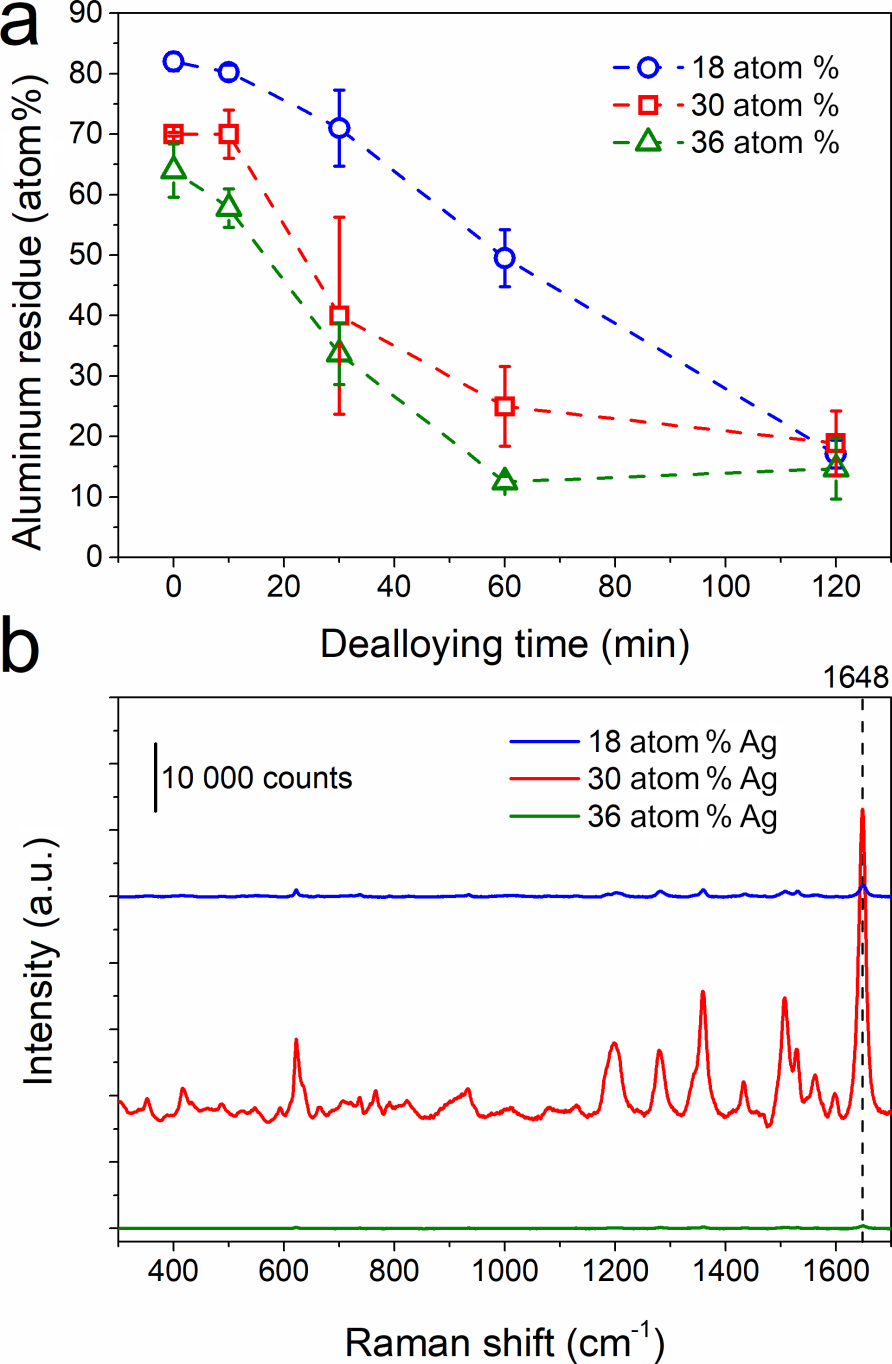
(a) Aluminium content evaluated by EDX for samples dealloyed in 1 wt % of HCl and for an initial Ag content of 18, 30, and 36 atom % in the film. (b) Raman spectra recorded after 24 h of incubation in a 10^−7^ mol·L^−1^ solution of RhB on a nanoporous silver film obtained after 60 min of dealloying in an HCl solution with an initial composition of 18, 30, and 38 atom %.

For a low amount of silver at the initial state (i.e., 18 atom %), small ligaments (≈25 ± 5 nm) can be observed on the surface (Figure 3a). Moreover, the structure is made of stacked layers as shown in the cross-section image (Figure 3d) with void and full layers. This is the result of the preferential dealloying of the aluminum-rich nanolayer as previously reported (Figure 2f). When increasing the amount of initial silver to 30 atom %, one can see an increase in the ligament size (≈52 ± 10 nm) and a typical nanoporous morphology made with interconnected ligaments (Figure 3e). Finally, when reaching 36 atom % of the initial silver content, the ligament size increases up to 114 ± 20 nm (Figure 3c) with the same structure observed through the thickness (Figure 3f). According to this observation, it seems that the kinetic of dealloying is slower for a lower amount of silver at the initial state. Indeed, small ligaments and the nanolayer structure are observed at an early stage of dealloying (Figure 2b and Figure 2f) compared to the largest ligaments at longer dealloying times (Figure 2d and Figure 2h). The EDX analysis of the samples dealloyed in HCl confirms the hypothesis related to the kinetic of dealloying (Figure 4a).

In Figure 4a it can be seen that the dealloying kinetics is faster for a higher amount of silver in the initial film with a faster decrease of aluminum content. However, this observation is not consistent with the previously reported studies on dealloying, revealing that the dealloying kinetics should be faster for samples with a lower amount of noble metals [24]. As previously shown for the case of the Ag–Al alloy dealloyed in HCl, the dealloying leads to the creation of a AgCl layer [41–42], more pronounced for a lower amount of Ag, which delays the dealloying [39,43]. This behavior can be seen for the sample AlAg30. The aluminum residue is stable after 10 min in an HCl solution and then drops to 25 atom % after 60 min in the etching solution. This behavior is associated to the creation of a AgCl layer at the surface of the structure at an early stage of dealloying. When the AgCl layer breaks down, then the HCl solution quickly spreads inside the structure and dissolves Al [43]. The largest standard deviation of the composition after 30 min of dealloying suggests that the dealloying kinetic is different on the substrate. Due to the initial morphology, the AgCl layer on every column breaks down after different times in the HCl solution. The discrepancy of kinetics observed here compared to that reported in the literature can be explained by the confinement effect which slows down the dealloying process in smaller pores since etching byproducts stay trapped and limit further dealloying of the structure. In other words, the extraction of AlCl_3_ formed during dealloying and confined in small pores is difficult since no solution agitation is applied, making the dealloying kinetics slower. As highlighted by the SEM images (Figure 3), the pores are smaller for low initial amounts of silver, thus the byproduct confinement effect is more likely to happen.

Then, we studied the influence of the initial Ag content and the dealloying time on the SERS detection properties. To reach this objective, Raman spectra of the RhB diluted at 10^−7^ mol·L^−1^ were recorded on the samples after different dealloying times and for the three selected initial Ag contents. No Raman signal was detected for the Ag–Al thin film before dealloying since the structure is mostly composed of aluminum. Likewise, no Raman signal originating from RhB was detected for all samples dealloyed for less than 60 min in HCl. The randomly organized small ligaments in the film and the rather high amount of aluminum residue did not allow for the formation of effective ‘hot spots’ to enhance the Raman signal of RhB. Figure 4b shows the RhB Raman spectra collected on samples dealloyed for 60 min. An intense Raman signal was recorded for the sample AlAg30 compared to that of the other conditions. Moreover, it can be noticed that the Raman signal for AlAg18 dealloyed for 60 min in HCl is slightly higher than that for the AlAg36 sample. The first explanation for this behavior can be associated to the increase in ligament size which hinders the Raman signal of RhB [44]. Indeed, the Raman signal of the AlAg36 sample is the weakest since the ligament size for this sample is the largest. To further confirm this hypothesis, the intensity of the RhB signal was recorded on nanoporous samples with the largest ligaments. In order to get nanoporous samples with larger ligaments, samples were dealloyed for longer times (i.e., 120 min, Supporting Information File 1, Figure S2) to reach a ligament size of 75 ± 10, 104 ± 14, and 153 ± 20 nm for AlAg18, AlAg30, and AlAg38, respectively. No Raman signal was recorded on these samples after immersion for 24 h in 10^−7^ mol·L^−1^ of RhB.

To understand the highest SERS performance of the AlAg30 sample dealloyed for 60 min, the composition of the surface of the film was probed by XPS. The SERS properties of a structure are driven by the presence of ‘hot spots’. These are highly sensitive to the composition of the surface since the presence of impurities can hinder the SERS properties [30]. The XPS composition of the surface of the samples dealloyed for 60 min in HCl and that for three selected Ag compositions is reported in Table 1.

**Table 1:** Surface composition evaluated by XPS of samples dealloyed for 60 min in 1 wt % HCl for different initial Ag content.

Composition (atom %)	C	O	Al	Cl	Ag

18 atom % of initial Ag	12.0	58.4	22.4	0.2	7.0
30 atom % of initial Ag	9.1	28.5	1.9	6.5	54.0
36 atom % of initial Ag	17.8	35.8	10.7	0.9	34.8

The sample showing the best SERS efficiency (i.e., AlAg30) is also the one with the highest concentration of Ag (54 atom %) and the lowest carbon concentration (9.1 atom %) on the surface. Besides the presence of chlorine originating from the dealloying process, the analysis of the Ag 3d peak (Supporting Information File 1, Figure S3 and Figure S4) reveals the presence of a loss peak indicating the metallic state of Ag. Therefore, no AgCl complex is observed. Moreover, a shift is observed for the Ag 3d peak towards higher binding energy values for the sample with low initial Ag content. This shift is related to the smaller size of the ligaments at a low Ag content as already reported for nanoparticles [45]. For the sample AlAg18, the amount of silver on the surface is the lowest (7 atom %) with a high amount of aluminum residue (22.4 atom %). As mentioned earlier, the kinetic of dealloying for this sample is slower than that for AlAg30 and AlAg36 which explains the high amount of aluminum on the surface. Besides the small size of ligaments, this low amount of silver leads to a lower SERS property for this sample. Conversely, the lower SERS efficiency for the sample AlAg36 dealloyed for 60 min can be associated to the presence of a higher carbon content on the surface (17.8 atom %) and the rather low amount of silver on the surface (34.8 atom %). As already reported, the presence of carbon on a metal surface induces hydrophobicity which can also affect the bonding with RhB molecules [30]. The carbon observed on the surface of the different samples is the result of the contamination of the substrate during wet etching and by the environment during storage [46]. Overall, the good performance of the AlAg30 sample dealloyed for 60 min in HCl results from a compromise between a small size of ligaments and the lowest amount of contamination together with the highest amount of silver on the surface.

#### Influence of the dealloying media

**Figure 5:**
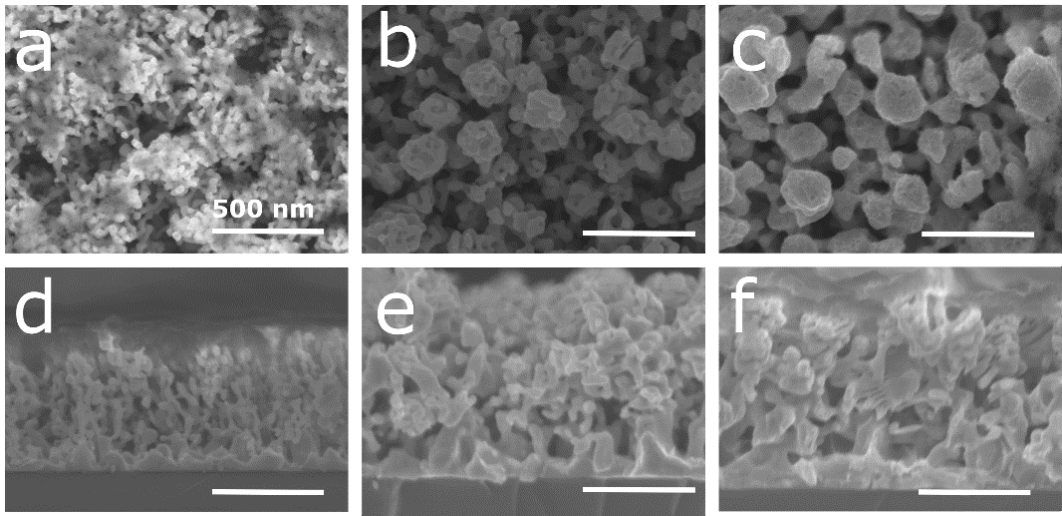
(a–c) Plan view and (d–f) cross-section SEM images of the Ag–Al alloy thin film dealloyed for 60 min in H_3_PO_4_ at 10 wt % with an initial composition of (a, d) 18, (b, e) 30, and (c, f) 36 atom % of Ag. Scale bar: 500 nm.

**Figure 6:**
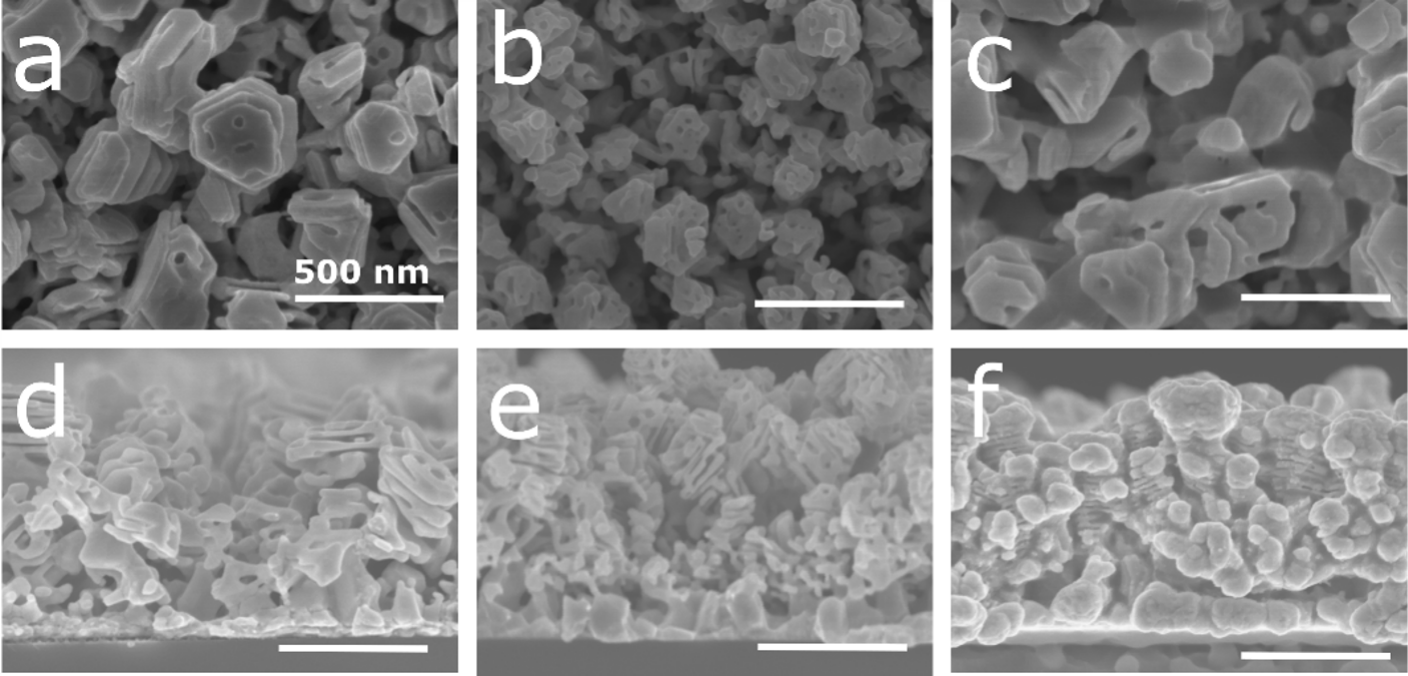
(a–c) Plan view and (d–f) cross-section SEM images of the Ag–Al alloy thin film dealloyed for 60 min in NaOH at 10 wt % with an initial composition of (a, d) 18, (b, e) 30, and (c, f) 36 atom % of Ag. Scale bar: 500 nm.

Finally, the influence of the dealloying media was studied. For this purpose, two other solutions were selected (i.e., H_3_PO_4_ and NaOH). Undoubtedly, anions play an important role during the dealloying process due to the intricate reaction between anions and Ag or Al [47–48]. It has been reported that during dealloying in HCl, the Cl^−^ ion accelerates the diffusion of Ag atoms, and the reaction between Cl^−^ and Al proceeds faster than that for other anions [47]. To complete the optimization of the SERS nanoporous silver substrate towards the detection of RhB, films with different silver compositions (i.e., 18, 30, and 36 atom %) were dealloyed using H_3_PO_4_ and NaOH. The SEM micrographs of the AlAg18, AlAg30, and AlAg36 samples dealloyed for 60 min in H_3_PO_4_ and NaOH are shown in Figure 5 and Figure 6, respectively.

Dealloying in H_3_PO_4_ (Figure 5) leads to the same morphology evolution for each composition in comparison to samples dealloyed in HCl (Figure 3). For the sample AlAg18 dealloyed in H_3_PO_4_ for 60 min, small ligaments (24 ± 4 nm) can be seen on the surface (Figure 5a). The initial structure made of dispersed nanocolumns is not visible in the cross-section image (Figure 5d). When increasing the initial concentration of silver to 30 atom %, the top of the columnar structure obtained at the initial state can be seen with the presence of small pores (Figure 5b). However, in the cross-section image, no columnar structure can be seen (Figure 5e). Finally, when reaching 36 atom % of silver at the initial state, the structure looks like a typical nanoporous structure with interconnected ligaments (Figure 5c and Figure 5f). A different behavior can be seen for samples dealloyed in NaOH. For AlAg18 dealloyed for 60 min, the structure exhibits the nanocolumn observed at the initial state (Figure 6a) with a stack of porous and empty layers (Figure 6d). For AlAg30 and AlAg36, the same nanoporous structure reported with dealloying in HCl and H_3_PO_4_ can be seen (Figure 6b–f). Despite the use of other dealloying media, the sample morphology is the same as that for AlAg30 dealloyed for 60 min in HCl.

**Figure 7:**
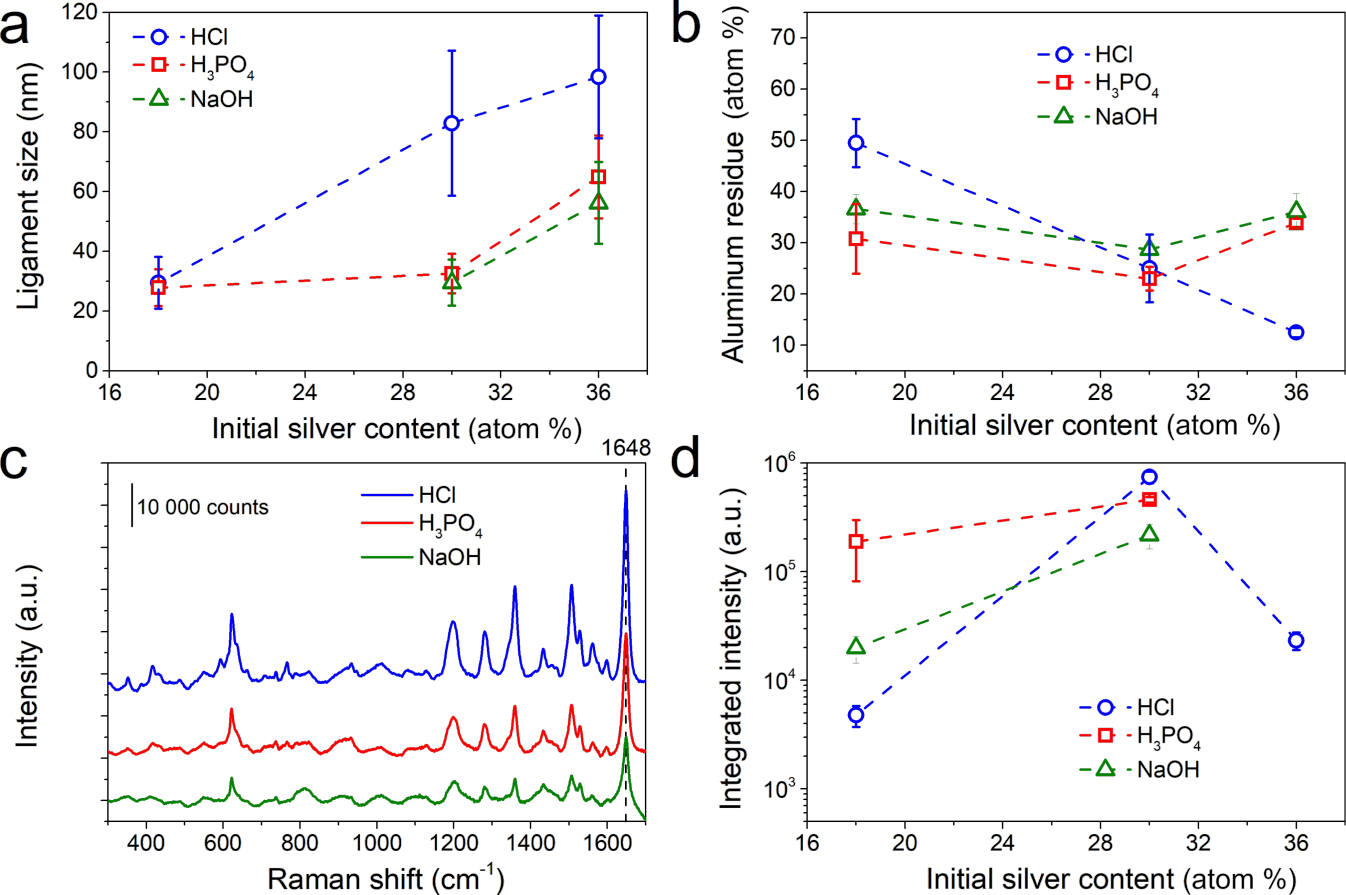
Evolution of the (a) ligament size and (b) aluminum residue for samples with different initial silver content at the initial state and dealloyed for 60 min in HCl (blue), H_3_PO_4_ (red), and NaOH (green). (c) Raman spectra recorded after 24 h of incubation in a 10^−7^ mol·L^−1^ RhB solution for Ag–Al samples with an initial amount of silver of 30 atom % and dealloyed for 60 min in (blue) HCl, (red) H_3_PO_4_, and (green) NaOH. (d) Comparison of the integrated intensity of the peak at 1648 cm^−1^ for samples with different initial silver content and dealloyed for 60 min in different solutions incubated for 24 h in 10^−7^ mol·L^−1^ of RhB.

Figure 7a highlights the variation in the ligament size for different initial silver contents in the thin film and in different etching media. The ligament size increases when the amount of initial silver in the film is increased for three dealloying media. It is important to mention here that for AlAg18 in NaOH no ligaments are seen in SEM images (Figure 6a). For AlAg18, the ligament size is similar (≈30 nm) for HCl and H_3_PO_4_ dealloying media. However, for higher initial silver contents, samples dealloyed in HCl reveal larger ligaments than those dealloyed in H_3_PO_4_ and NaOH. This observation can be related to the fast diffusion of Ag promoted by Cl^−^ ions [47]. Figure 7b displays the content of aluminum residue in samples after dealloying probed by EDX. Two different behaviors can be observed: for the HCl medium, the aluminum residue content decreases when the initial silver content is increased whereas for H_3_PO_4_ and NaOH, no change in the aluminum residue content is observed. For H_3_PO_4_ and NaOH, the dealloying proceeds at the same rate for all samples until the formation of a protective layer containing silver, which protects the aluminum from further etching. Conversely, in the case of dealloying in HCl, the reaction with silver led to the creation of a AgCl layer protecting the aluminum etching until the formation of soluble [AgCl_2_]^−^ complexes. The breakdown of the protective layer allows for the etching of more quantities of aluminum compared to those after dealloying in H_3_PO_4_ and NaOH [43]. Therefore, for a high initial silver content in the thin film, the aluminum residue is lower for the sample dealloyed in HCl. On the other hand, a higher amount of aluminum residue after etching in HCl for the lowest amount of silver at the initial state might be due to the confinement effect discussed before. This affects the dealloying kinetics in a different way according to the initial silver content since smaller pores are observed for a lower amount of initial silver content. This effect is more pronounced for samples dealloyed in HCl since the Cl^−^ ions accelerate the diffusion of the silver atoms and promote dealloying [47]. After 120 min of dealloying, the aluminum residue drops to near 17 atom % for HCl and H_3_PO_4_ for three different initial silver contents (Supporting Information File 1, Figure S5). For NaOH, the film completely delaminates after 120 min of dealloying, so the EDX analysis was not possible.

As previously mentioned, surface composition drives SERS properties. Thus, the surface composition of the AlAg30 samples dealloyed for 60 min in NaOH and in H_3_PO_4_ was evaluated by XPS (Table 2).

**Table 2:** Surface composition of nanoporous silver thin films with 30 atom % of initial Ag content and dealloyed for 60 min in different dealloying media evaluated by XPS.

Composition (atom %)	C	O	Al	Ag	P

30 wt % NaOH	22.2	35.6	11.6	30.6	–
10 wt % H_3_PO_4_	11.4	51.1	15.6	20	1.9

As for the sample dealloyed in HCl, the Ag 3d peak recorded on the sample dealloyed in H_3_PO_4_ reveals the presence of metallic silver at the surface of the nanoporous structure (Supporting Information File 1, Figure S6). Moreover, the presence of 2 atom % of phosphorous coming from the etching solution can be noticed. The influence of the dealloying media on the SERS efficiency was probed for the detection of RhB (Figure 7c and Figure 7d). The Raman spectra of AlAg30 samples dealloyed in different media are displayed in Figure 7c. The integrated intensity of the peak at 1648 cm^−1^ is highlighted in Figure 7d. For a low initial amount of silver (i.e., 18 atom %), besides a similar ligament size for all samples, the SERS efficiency is higher for H_3_PO_4_. This higher efficiency can be associated with the lowest amount of aluminum residue evaluated by EDX. The next best SERS efficiency is observed for AlAg30 samples. Even though all samples have similar amounts of aluminum residue independently of the dealloying media, the best efficiency is reported for HCl. In this case, the chemical composition of the surface (i.e., high amount of silver and low amount of carbon) might explain the best SERS efficiency. Finally, for a higher initial silver content (i.e., 36 atom %), no SERS signal was detected for samples dealloyed in H_3_PO_4_ and NaOH, and a drop in the intensity of the Raman signal was observed for samples dealloyed in HCl. Larger ligaments together with a high amount of carbon at the surface can explain the decrease or hindrance in the SERS signal for samples with a high silver content.

### Detection limit

**Figure 8:**
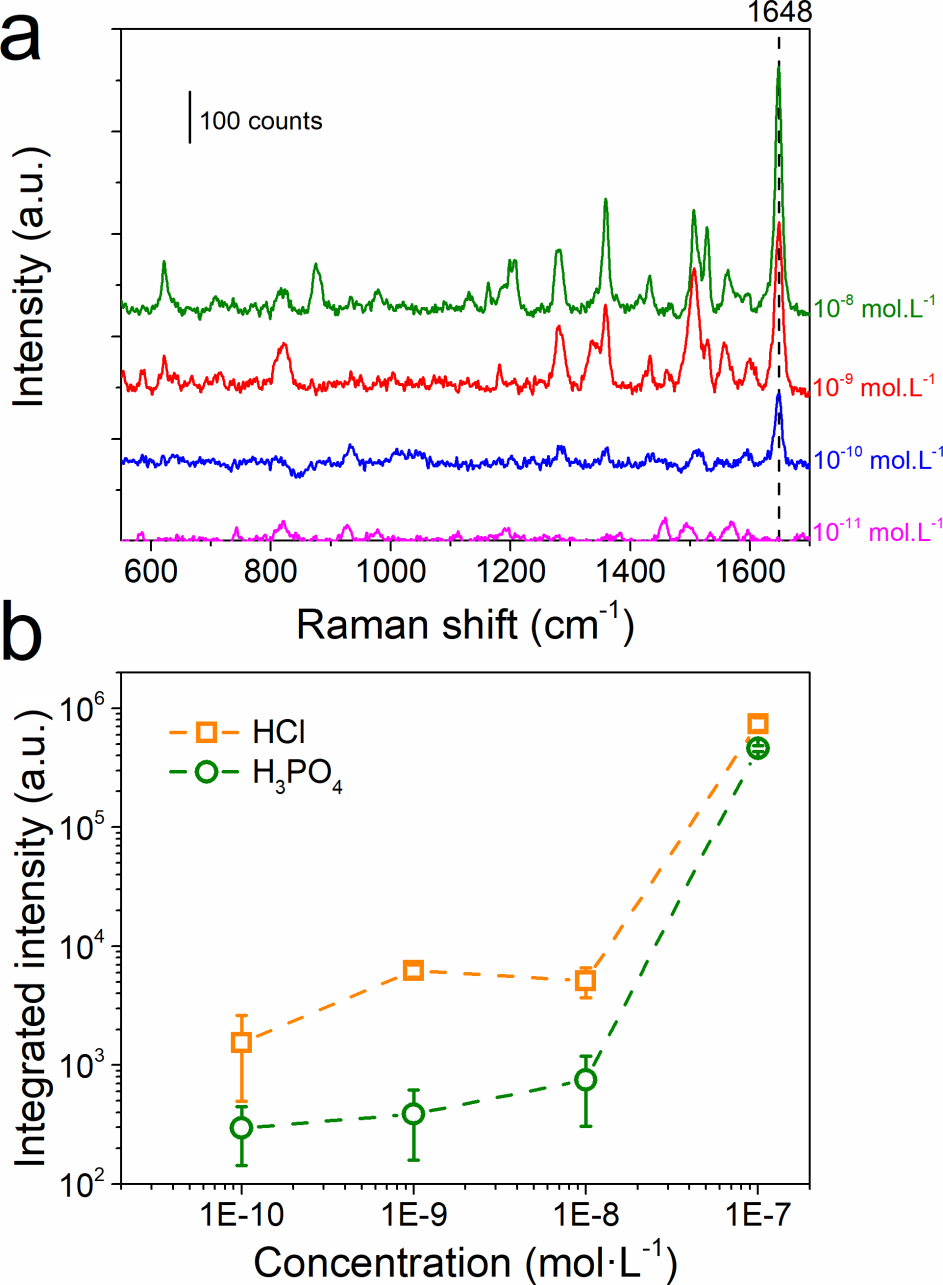
(a) Raman spectra recorded after 24 h of incubation in a RhB solution for different concentrations on a sample with an initial amount of silver of 30 atom % and dealloyed for 60 min in 1 wt % of HCl. (b) Comparison of the evolution of the integrated peak at 1648 cm^−1^ for different concentrations of RhB for samples with 30 atom % of silver at the initial state and dealloyed for 60 min in (orange) 1 wt % of HCl and (green) 10 wt % of H_3_PO_4_.

SERS-based sensors are mostly built to detect low concentration of molecules in a solution. Therefore, RhB solutions were diluted in order to reach the limit for which the signal of the molecule cannot be seen, corresponding to the limit of detection (LoD) of our material (Figure 8a).

For this part, AlAg30 thin films dealloyed for 60 min were selected since they present the highest Raman intensity of RhB at 10^−7^ mol·L^−1^ (Figure 8b). For the sake of comparison, samples dealloyed in HCl and H_3_PO_4_ were analyzed. Figure 8a displayed Raman spectra measured on the thin film dealloyed with HCl. The Raman signal of RhB strongly decreases when the RhB concentration is decreased from 10^−7^ to 10^−8^ mol·L^−1^. Then a plateau is observed until the Raman signal for a RhB concentration of 10^−11^ mol·L^−1^ is complete vanished. The behavior of the SERS response is comparable to that reported in the literature and can be related to the adsorption isotherm curve of RhB [49–50]. The plateau in the SERS response observed between 10^−8^ and 10^−10^ mol·L^−1^ can be assigned to either the saturation of the chemical sites (i.e., no supplementary absorption is made) or to the fact that RhB continued to be absorbed outside of the hot spot [6,51]. Comparing the integrated intensity of the RhB peak, it can be observed that the intensity of the signal is lower for the sample dealloyed in H_3_PO_4_ than that for the sample dealloyed in HCl. Besides a lower intensity, the detection limit for both substrates is below 10^-10^ mol·L^−1^. The LoD reported here is lower than the maximum allowable residue limit for RhB in food fixed by the European Union standard (1.09 × 10^−9^ mol·L^−1^) [52]. Besides a lower LoD for RhB reported for complex nanostructures with grafted molecules (10^−12^ mol·L^−1^) [53], or prepared from colloidal solutions (10^−13^ mol·L^−1^) [8,54], the approach reported here allows for an easy synthesis of a rather high sensible platform. Alternatively, more simple approaches for the synthesis of SERS-based sensors, such as direct physical vapor deposition (PVD) coating of natural micro- or nanostructured materials have been reported. For example, the use of Taro leaves or rose petals as substrates for silver PVD coating leads to a SERS detection limit down to 10^−8^ mol·L^−1^ and 10^−9^ mol·L^−1^, respectively, for rhodamine 6G (R6G) [55–56]. Moreover, the use of silver-coated paper as a SERS substrate reveals a detection limit down to 10^−10^ mol·L^−1^ for R6G [57]. Besides an easy synthesis and a good detection limit, these substrates cannot be cleaned and reused. In the case of nanoporous silver, the reusability of the structure for SERS detection has already been reported [1].

## Conclusion

The development of an easy strategy to engineer efficient SERS platforms using nanoporous silver is reported for the detection of RhB. The synthesis approach relies on the structuration of a Ag–Al alloy thin film deposited by magnetron sputtering followed by dealloying. The structuration at the microscale was obtained by taking advantage of abnormal growth of the Ag–Al alloy thin film together with the shadowing effect to form dispersed columnar thin films. The subsequent dealloying leads to film structuration at the nanoscale due to porosity formation. The influence of three different dealloying solutions (i.e., NaOH, HCl, and H_3_PO_4_) on the SERS properties of the nanoporous structure is reported. Following the characterization of the surface, the structure and surface composition were correlated with SERS properties. Following the optimization of the thin film structuration, a limit of detection of 10^−10^ mol·L^−1^ is demonstrated. The high sensibility and the straightforward synthesis together with the easy operability of this platform make it very promising for practical applications such as detection of low concentrations of pollutants or biomolecules.

## Experimental

### Synthesis of Ag–Al thin films

The synthesis approach of Ag–Al thin films and the dealloying procedure were adapted from [39]. The Ag–Al thin films were deposited by DC magnetron co-sputtering in pure argon plasma of a Ag target (diameter: 50.8 mm; purity: 99.99%) and an Al target (diameter: 50.8 mm; purity: 99.99%) placed in a confocal geometry. The distance between the targets and the substrate was 100 mm and the angle between the magnetron source axis and the normal to the substrate was 30°. The substrate was silicon. Prior to each deposition, a 50 nm Ag adhesion layer was grown by magnetron sputtering. While the power on the aluminum target was fixed to 150 W, the power on the silver target was tuned to 25, 50, and 75 W to create thin films with 18, 30, and 36 atom % of silver, respectively. The deposition was performed over a substrate rotating at 1.6 rpm. For all depositions the base pressure was less than 5 × 10^−6^ Torr and the deposition pressure was fixed to 5 × 10^−3^ Torr. The deposition time was fixed to 12 min to reach a thin film with a thickness of 700 ± 40 nm.

### Dealloying of Ag–Al thin films

The dealloying of Ag–Al thin films was carried out in HCl (1 wt %), NaOH (30 wt %), and H_3_PO_4_ (10 wt %). The samples were immersed for the desired time and dipped into deionized water for 20 min to stop the dealloying process and to ensure a good cleaning of the samples.

### Characterization

Scanning electron microscopy micrographs were recorded using a HITACHI STEM-FEG with an acceleration voltage of 5 kV. The images were treated with the ImageJ software [58] to assess the size of the ligaments. Each ligament size was measured 50 times. The measurements were performed in different SEM images taken in different positions over the sample. The chemical composition of the films was determined by EDX with an acceleration voltage of 10 kV. The measurements were performed in five positions over a sample and on two samples made in the same conditions. To evaluate the surface composition and oxidation state, XPS was used. The XPS measurements were carried out on a PHI 5000 VersaProbe using a monochromatic Al Kα X-ray source (1486.6 eV). The high-resolution spectra were recorded with a pass energy of 23 eV.

### Surface-enhanced Raman scattering measurements

Raman measurements were performed with a Senterra Bruker micro-Raman spectrometer using a 533 nm excitation laser line with an acquisition time of 10 s. The power was fixed to 0.2 mW and focused on the sample with a ×50 objective. Rhodamine B was chosen as the SERS probe molecule. Prior to experiments, porous silver samples were dipped into ultrapure water and dried. The samples were then immersed for 24 h into a solution of ultrapure water solution containing RhB with concentration values varying from 10^−7^ to 10^−11^ mol·L^−1^, enabling the RhB molecules to be absorbed on the surface. This procedure was already used in our previous studies [6]. The samples were then dried in air prior to SERS measurements. Each analysis was carried out on three different samples made in the same conditions and at three different areas for each sample to get the standard deviation. In order to test the reproducibility of a given surface, the analysis was performed in nine different areas of the same sample, and the relative standard deviation value for the integrated intensity was 16% (Supporting Information File 1, Figure S7).

## Supporting Information

File 1 Additional figures.
