# Supplementary material for: Combining physical vapor deposition structuration with dealloying for the creation of a highly efficient SERS platform
Source: Beilstein J Nanotechnol. 2023 Jan 11;14:83–94. doi: 10.3762/bjnano.14.10 (PMC9843236; doi:10.3762/bjnano.14.10)
Supplement: File 1 — Additional figures. [file Beilstein_J_Nanotechnol-14-83-s001.pdf]

BEILSTEIN JOURNAL OF NANOTECHNOLOGY

Supporting Information

for

Combining physical vapor deposition structuration with dealloying for the creation of a highly efficient SERS platform

Adrien Chauvin, Walter Puglisi, Damien Thiry, Cristina Satriano, Rony Snyders and Carla Bittencourt

Beilstein J. Nanotechnol. **2023,** *14,* 83–94. doi:10.3762/bjnano.14.10

Additional figures

License and Terms: This is a supporting information file under the terms of the Creative Commons Attribution License (https://creativecommons.org/licenses/by/4.0). Please note that the reuse, redistribution and reproduction in particular requires that the author(s) and source are credited and that individual graphics may be subject to special legal provisions.
The license is subject to the *Beilstein Journal of Nanotechnology* terms and conditions: (https://www.beilstein-journals.org/bjnano/terms)

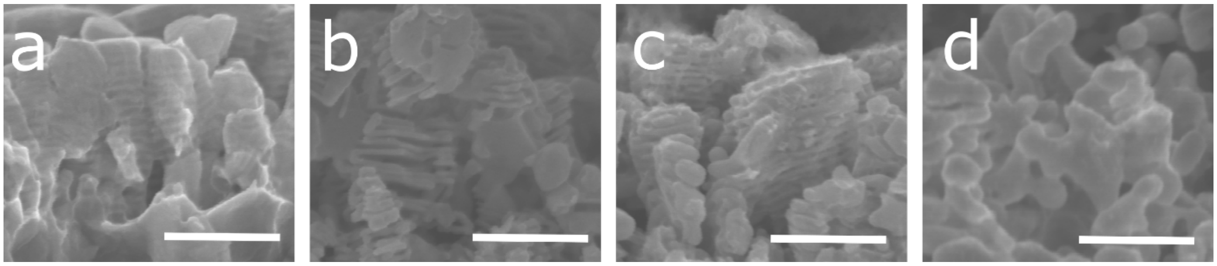

Figure S1: High magnification of cross-section SEM images of the (a) initial Ag–Al thin film with 30 atom % of Ag and dealloyed for (b) 10, (c) 30, and (d) 60 min. Scale bar: 250 nm. The high magnification of the cross sections highlights the nanolayer morphological structure and behavior throughout the dealloying process.

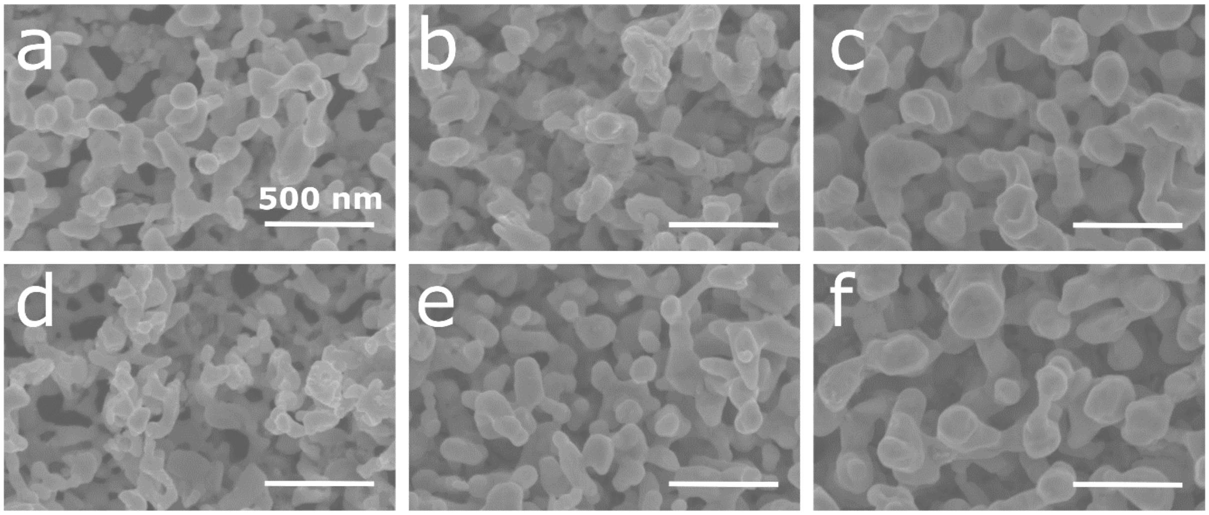

Figure S2: Plan view SEM images of Ag–Al thin films dealloyed for 120 min in (a–c) HCl and (d–f) H_3PO_4 for a sample with an initial composition of (a and d) 18, (b and e) 30, and (c and f) 36 atom % of Ag. Scale bar: 500 nm. Depending on the initial silver content, the morphology after dealloying for 120 min in HCl and H_3PO_4 does not change. The ligament size increases when the initial silver content is increased.

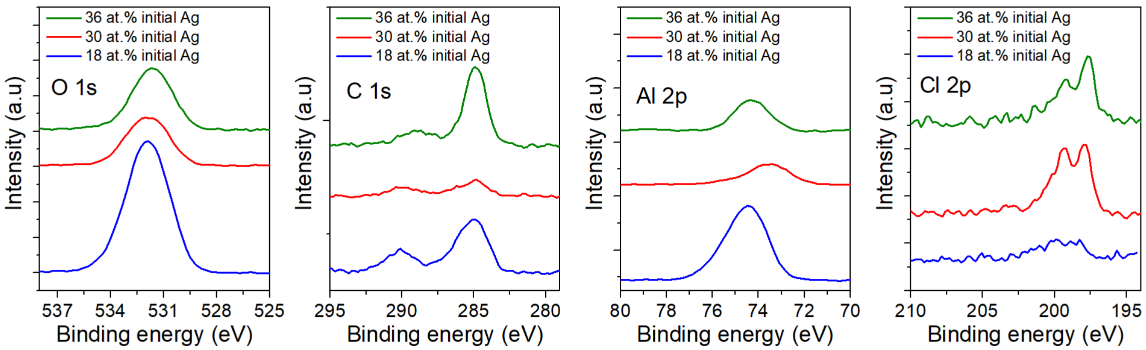

Figure S3: XPS (O 1s, C 1s, Al 2p, and Cl 2p) spectra recorded on a Ag–Al alloy thin film dealloyed for 60 min in an HCl solution (1 wt %) with an initial composition of 18, 30, and 36 atom % of Ag.

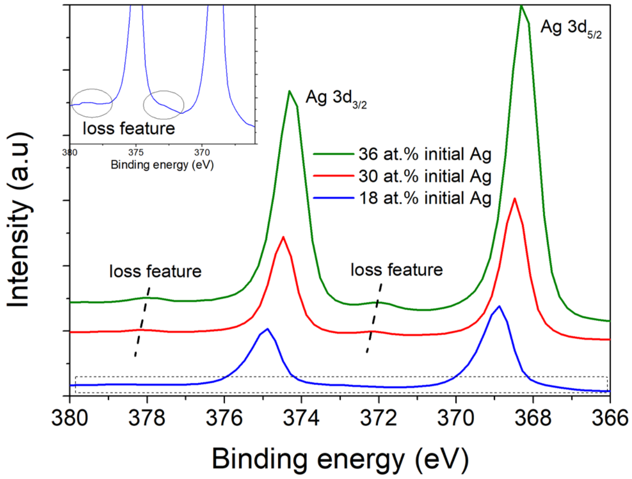

Figure S4: XPS Ag 3d recorded on a Ag–Al alloy thin film dealloyed for 60 min in an HCl solution (1 wt %) with an initial composition of 18, 30, and 36 atom % of Ag. The inset is a zoom of the dotted area corresponding to the sample with 18 atom % of initial Ag. All samples after dealloying for 60 min highlight the loss feature characteristic of metallic silver.

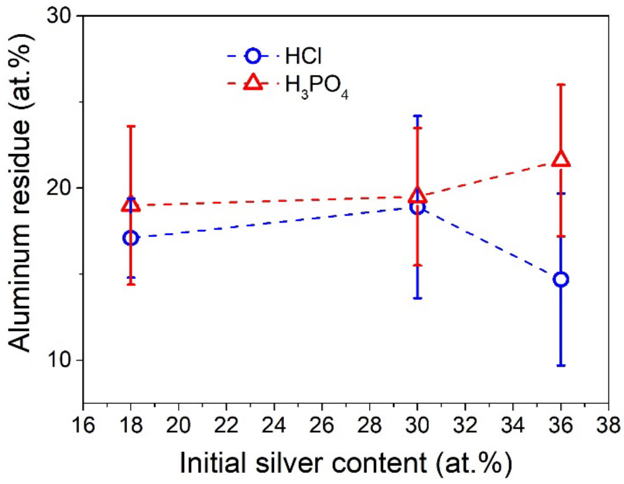

Figure S5: Aluminum residue probed by EDX of the Ag–Al alloy thin film dealloyed for 120 min in a H_3PO_4 solution (10 wt %) and in an HCl solution (1 wt %) with an initial composition of 18, 30, and 36 atom % of Ag.

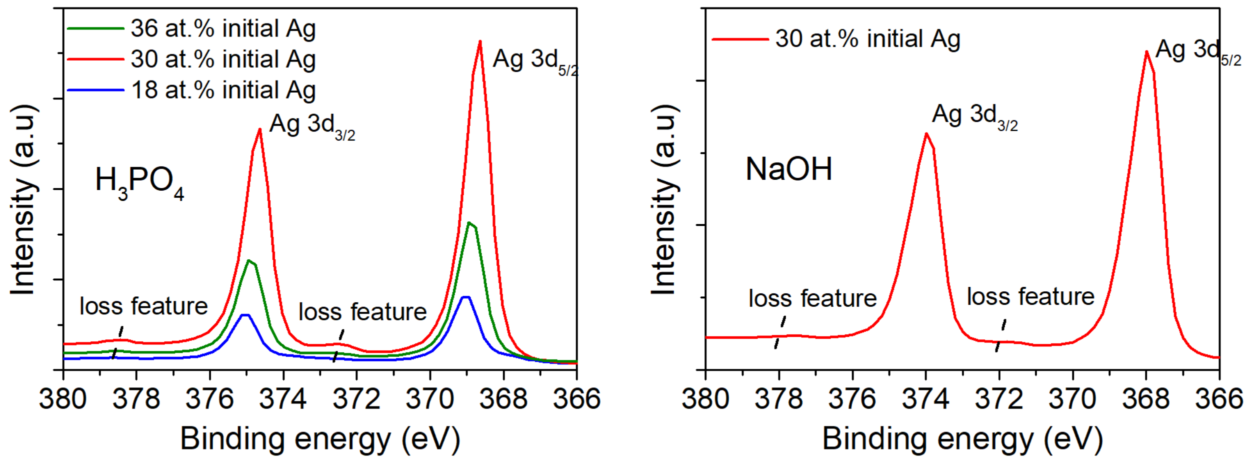

Figure S6: XPS analysis of the Ag 3d peak of the Ag/Al alloy thin film dealloyed during 60 min in (left) H_3PO_4 solution at 10 wt % with an initial composition of 18, 30, and 36 atom % of Ag and in (right) a NaOH solution at 30 wt % with an initial composition of 30 atom % of Ag.

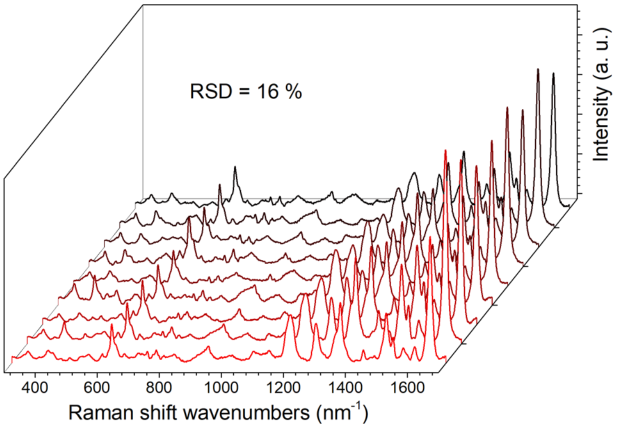

Figure S7: Raman spectra of RhB diluted at 10^{-7} mol·L^{-1} and recorded in nine different places over the nanoporous silver surface made by dealloying an Ag–Al alloy film with 30 atom % of Ag at the initial state and dealloyed for 60 min in a HCl solution at 1 wt %. Following these analyses, the relative standard deviation (RSD) for the intensity value of the peak at 1648 cm^{-1} was evaluated to be 16%.
